# Unilateral Cervical Lymphadenopathy due to* Cladosporium oxysporum*: A Case Report and Review of the Literature

**DOI:** 10.1155/2017/5036514

**Published:** 2017-10-25

**Authors:** R. D. Jayasinghe, W. A. M. U. L. Abeysinghe, P. I. Jayasekara, Y. S. Mohomed, B. S. M. S. Siriwardena

**Affiliations:** ^1^Department of Oral Medicine and Periodontology, Faculty of Dental Sciences, University of Peradeniya, Peradeniya, Sri Lanka; ^2^Department of Oral Pathology, Faculty of Dental Sciences, University of Peradeniya, Peradeniya, Sri Lanka; ^3^Department of Mycology, Medical Research Institute, Colombo, Sri Lanka; ^4^General Hospital, Polonnaruwa, Sri Lanka

## Abstract

Phaeohyphomycosis is a fungal infection caused by Dermatiacae group of fungi, by* Cladosporium spp. *The term phaeohyphomycosis was introduced by Ajello et al. in 1974 to designate infections by brown pigmented filamentous fungi.* Cladosporium oxysporum *is a very rare etiological agent in humans. Phaeohyphomycosis of the cervical lymph node in an immunocompetent individual is a very rare clinical entity. To the best of our knowledge we report the first case of phaeohyphomycosis caused by* Cladosporium oxysporum* in the absence of other systemic manifestations in a 16-year-old male.

## 1. Introduction

The spectrum of mycotic disease continues to expand and the field of medical mycology has become a challenging study of infections caused by diverse array of opportunistic fungi. The term phaeohyphomycosis was introduced by Ajello et al. in 1974 [1] to designate infections by pigmented filamentous fungi that contain melanin in their walls and these organisms can be found in wood and decomposing plants. Though it is rare in humans, they can cause life threatening infections in both immunocompromised and immunocompetent individuals. Cutaneous and corneal manifestations are present in healthy individuals and both cutaneous and systemic phaeohyphomycosis occur in immunocompromised patients [2].

The clinical syndromes caused by melanized fungi are differentiated based on histologic findings into eumycetoma, chromoblastomycosis, and phaeohyphomycosis. Eumycetoma is a deep tissue infection, usually of the lower extremities, characterized by the presence of mycotic granules [3]. It is associated with a relatively small group of fungi. Chromoblastomycosis is caused primarily by a few species of fungi that produce characteristic sclerotic bodies in tissue and is usually seen in tropical areas [3]. Pigmented hyphae are the hallmark of phaeohyphomycosis rather than the round intermediate bodies of chromoblastomycosis.* Exophiala*,* Bipolaris*,* Curvularia*,* Lecythophora*,* Dactylaria*,* Phialophora*,* Exserohilum*,* Alternaria*, and* Cladosporium* are species of several genera of fungi that have been recognized as agents of phaeohyphomycosis [4, 5].

The species of* Cladosporium* implicated in human infections are* C. cladosporioides*,* C. herbarum, C. sphaerospermum, C. elatum, and C. oxysporum* [5, 6]. These hyphomycetes have been found as causative agents for infections of the central nervous system, lung and liver infections, keratitis, and dental granuloma [7]. Compared to chromoblastomycosis, skin infections are rare in phaeohyphomycosis. Skin infections caused by* Cladosporium oxysporum* is even more rarer in phaeohyphomycosis [7, 8].

Phaeohyphomycosis of the cervical lymph node in an immunocompetent individual is a very rare clinical entity. To the best of our knowledge we report the first case of phaeohyphomycosis caused by* Cladosporium oxysporum* in the absence of other systemic manifestations in a 16-year-old male.

## 2. Case Report

**Figure 1:**
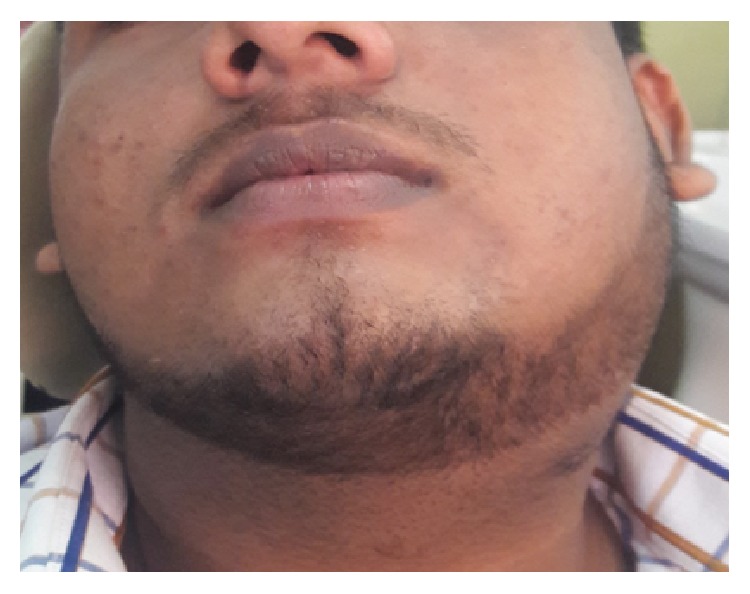
L/S neck swelling due to lymphadenopathy.

**Figure 2:**
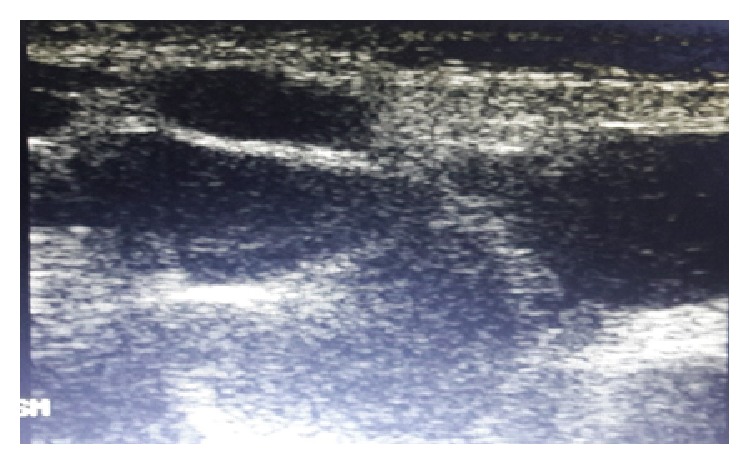
Ultrasound scan of the neck; multiple enlarged cervical lymph nodes at the levels of I-B, II, III, and IV. Largest at level II was 3.7 cm × 2.0 cm in size.

A 16-year-old well built, otherwise healthy boy presented with a 3-month history of a painless swelling over his left neck just below the angle of the mandible (Figure 1). On palpation there were multiple enlarged cervical lymph nodes. Ultrasound scan revealed multiple enlarged cervical lymph nodes of levels I-B, II, III, and IV and largest was at level II with 3.7 cm × 2.0 cm in size (Figure 2). The cytology report following fine needle aspiration showed numerous eosinophils admixed with lymphocytes and occasional neutrophils. Blood report revealed a marked eosinophilia. Considering these findings, Kimura's disease was suspected with a remote possibility of lymphoma in mind; largest lymph node was removed surgically. Consent was given by the parents.

**Figure 3:**
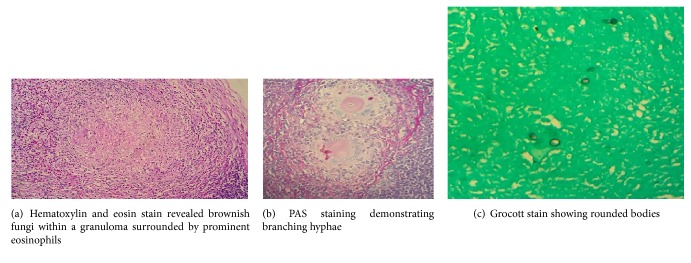


**Figure 4:**
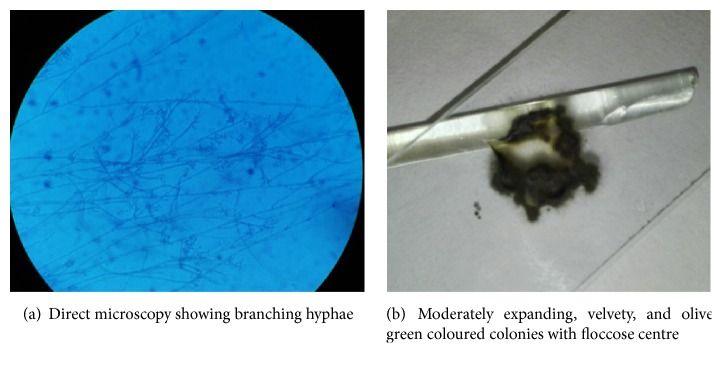


Histopathologically the lymph node showed completely effaced architecture with multiple granulomas in the background of numerous eosinophils. Faint germinal centres were also noted. There were brown coloured small rounded bodies and hyphae within giant cells (Figure 3(a)). Periodic Acid Schiff (PAS) and Grocott special stains highlighted the presence of thick walled round and filamentous fungi confined to granulomas but within and outside giant cells (Figures 3(b) and 3(c)). Direct microscopy was done using 10% Potassium Hydroxide (KOH) and we were able to see fungal hyphae. Cultures were obtained on Sabouraud dextrose agar supplemented with chloramphenicol and cyclohexamide (to suppress bacteria and environmental fungi) and incubated at 26°C and 37°C. Moderately expanding, velvety, and olive green coloured colonies with floccose centre were yielded and confirmed the organism as* Cladosporium oxysporum* and then the final diagnosis was phaeohyphomycosis (Figures 4(a) and 4(b)). Antifungal treatment was started with 200 mg of Itraconazole twice a day. Itra capsules were used as we do not have suspension in Sri Lanka. Therefore, the bioavailability is less compared to suspension. Further it is necessary to state that we do not have facilities to do therapeutic drug level monitoring which is mandatory to do while the patient is on Itra. The problem related is that it is difficult to optimize the dose. Although the swelling has not subsided completely, the patient is currently under follow-up.

## 3. Discussion

Phaeohyphomycosis is a fungal infection caused by Dermatiacae group of fungi by* Cladosporium *spp. [9]. They are pigmented moulds found in soil and in plants and can give rise to three clinical entities such as eumycetoma, chromoblastomycosis, and phaeohyphomycosis. This genus comprises more than 30 species and about 10 species were mostly isolated so far including* Cladosporium oxysporum.* In the saprophytic phase it forms pigmented septate hyphae ending with conidia in contrast with parasitic phase which can be observed in tissues as brownish structures ranging from yeast-like to hyphae [9]. It is a very rare etiological agent of keratitis and phaeohyphomycosis (cutaneous) and we found only 2 cases in the English literature (Table 1). Some authors suggested that lesions of subcutaneous type clinically present as nodules or abscesses with a slight tendency towards lymphatic or haematogenous dissemination [6, 8]. A case reported by Gugnani et al. [8] indicated that the patient was a farmer and had a history of trauma to the skin in contrast with the present case. Although the present case gave no history of any kind of laceration, there may be an unnoticed damage to the skin of the affected area. Another possible route is environmental exposure.

Histologically all Dermatiacae fungi show melanin pigments and if it is very weak to observe in routine haematoxyllin and eosin stained sections, it can be highlighted by Fontana-Masson staining. However, caution should be exercised when interpreting this special staining, because many* Aspergillus* spp., some* Mucorales* genera, and* Trichosporon* can also show positive staining [10]. In chromoblastomycosis there are characteristic sclerotic bodies which can be single or clusters and they are also called “copper penny lesions” which may show internal septation. Surface epithelium shows pseudoepitheliomatous hyperplasia and hyperkeratosis. Phaeohyphomycosis produces granulomatous inflammation with abundant giant cell with necrosis. Yeast-like structures and hyphae can be found throughout the lesion. Mycetomas produce characteristic black grains and they are histologically interwoven. There are white grains produced by bacteria. The different pigmented fungi cannot be distinguished from one another and culture is required for confirmation.

## Figures and Tables

**Table 1:** Cases reported in the literature.

Reference	Age/sex	Site	Duration	Underlying condition
Romano et al. (1999) [7]	66 F	Right leg (cutaneous) papulonodular lesion	1 year	Cushing syndrome
Gugnani et al. (2006) [8]	30 F	Left foot near ankle, multiple coalescing ulcers	3 years	Immunocompetent
Present case	16 M	Cervical lymph nodes of levels I–IV	3 months	Immunocompetent
